# Recent Progress Toward Hydrogen Medicine: Potential of Molecular Hydrogen for Preventive and Therapeutic Applications

**DOI:** 10.2174/138161211797052664

**Published:** 2011-07

**Authors:** Shigeo Ohta

**Affiliations:** Department of Biochemistry and Cell Biology, Institute of Development and Aging Sciences, Graduate School of Medicine, Nippon Medical School

**Keywords:** Anti-inflammation, antioxidant, hydrogen medicine, medical gas, mitochondria, oxidative stress, ischemia-reperfusion, ROS.

## Abstract

Persistent oxidative stress is one of the major causes of most lifestyle-related diseases, cancer and the aging process. Acute oxidative stress directly causes serious damage to tissues. Despite the clinical importance of oxidative damage, antioxidants have been of limited therapeutic success. We have proposed that molecular hydrogen (H_2_) has potential as a “novel” antioxidant in preventive and therapeutic applications [Ohsawa *et al*., Nat Med. 2007: 13; 688-94]. H_2_ has a number of advantages as a potential antioxidant: H_2_ rapidly diffuses into tissues and cells, and it is mild enough neither to disturb metabolic redox reactions nor to affect reactive oxygen species (ROS) that function in cell signaling, thereby, there should be little adverse effects of consuming H_2_. There are several methods to ingest or consume H_2_, including inhaling hydrogen gas, drinking H_2_-dissolved water (hydrogen water), taking a hydrogen bath, injecting H_2_-dissolved saline (hydrogen saline), dropping hydrogen saline onto the eye, and increasing the production of intestinal H_2_ by bacteria. Since the publication of the first H_2_ paper in *Nature Medicine* in 2007, the biological effects of H_2_ have been confirmed by the publication of more than 38 diseases, physiological states and clinical tests in leading biological/medical journals, and several groups have started clinical examinations. Moreover, H_2_ shows not only effects against oxidative stress, but also various anti-inflammatory and anti-allergic effects. H_2_ regulates various gene expressions and protein-phosphorylations, though the molecular mechanisms underlying the marked effects of very small amounts of H_2_ remain elusive.

## 1 INTRODUCTION

Oxidative stress arises from the strong cellular oxidizing potential of excess reactive oxygen species (ROS) [1]. Acute oxidative stress arises from a variety of situations, including ischemia reperfusion [2]. Persistent oxidative stress is widely accepted as one of the causes of most lifestyle-related diseases, cancer and the aging process [3-7]; however, many antioxidant supplements could not prevent cancer, myocardial farction and atherosclerosis, but rather conversely increase mortality [8-11]; thus, it is very important to be aware of side effects when developing an effective antioxidant for the prevention of oxidative stress-related diseases.

We found that molecular hydrogen (H_2_) has roles as a “novel” antioxidant in preventive and therapeutic applications [12]. H_2_ has advantages as a potential antioxidant without adverse effects: it is mild enough neither to disturb metabolic redox reactions nor to affect ROS, which function in cell signaling [13-15] and has favorable distribution characteristics in its own physical ability to penetrate biomembranes and diffuse through barriers into cellular components.

Here, we review the recent progress toward therapeutic and preventive applications of H_2_ in widespread fields. 

## 2 ROS AS ONE OF THE MAJOR CAUSES OF ACUTE AND CHRONIC DISEASES

### 2.1 Persistent Oxidative Stress 

ROS are generated inside the body throughout our daily lives, such as during hard exercise, smoking, exposure to ultraviolet rays or air pollution, aging, physical or psychological stress, and so on [16-19]. Inside every aerobic organism, ROS are generated when breathing consumes oxygen. 

As the first step in generating persistent ROS, the majority of superoxide anion radicals (●O_2_^-^) are generated in mitochondria by electron leakage from the electron transport chain [3, 7 20, 21]. Superoxide dismutase converts to hydrogen peroxide (H_2_O_2_), which is metabolized by glutathione peroxidase or catalase to generate water (H_2_O). Highly reactive hydroxyl radicals (●OH) are generated from H_2_O_2 _via the Fenton or Weise reaction in the presence of catalytically active metals, such as Fe^2+^ and Cu^+^ [22]; therefore, manipulation of the genes involved in anti-oxidation prolonged the lifespan or prevented disease models [23-27]. 

These ROSs are generated under the condition of excessively high membrane potential to leak electrons from the electron transport chain [28]. In fact, uncoupling proteins control the membrane potential to suppress the production of ROS and then consequently to repress diabetes [29-31]. 

Mitochondrial aldehyde hydydrogenase 2 (ALDH2) functions as a protector against oxidative stress by detoxifying cytotoxic aldehydes, such as 4-hydroxy-2-nonenal [4, 5, 32]. Thus, a defect of ALDH2 sufficiently induces phenotypes of age-dependent dementia by accumulating such cytotoxic aldehydes [32]. Paradoxically, such aldehydes stimulate protective systems against oxidative stress [33]. Thus, oxidative stress has two faces, to damage tissues and to enhance protective systems. 

### 2.2 Acute Oxidative Stress

Acute oxidative stress arises from various different situations: inflammation, cardiac or cerebral infarction, organ transplantation, heavy exercise, cessation of operative bleeding, and others [2, 34, 35]. In many cases, ischemia reperfusion is a critical cause to raise acute oxidative stress. In myocardial infarction, the accelerated generation of ROS by reperfusion of the ischemic myocardium is a potential mediator of reperfusion injury [36-39]. During myocardial reperfusion, ●O_2_^-^ is generated within the injured mitochondria via electron leakage from the electron transport chain. ●O_2_^-^ converts to H_2_O_2_, and highly reactive ●OH is generated from H_2_O_2_ as mentioned [22, 40].

These ROS mediate myocardial injury by inducing mitochondrial permeability transition pore (PTP) opening, causing a loss of mitochondrial membrane potential, and leading to mitochondrial swelling with membrane rupture [41]. Many attempts have been made to inhibit ROS production to limit the extent of reperfusion injury. The administration of ROS scavengers at the time of reperfusion has produced conflicting results that can be partially explained by the dual role of ROS in ischemia-reperfusion hearts [42, 43]. The majority of detrimental effects associated with lethal reperfusion injury are attributed to ●OH. By comparison, ●O_2_^-^ and H_2_O_2_ have less oxidative energy and, paradoxically, are implicated as crucial signaling components in the establishment of tolerance to oxidative stress [44, 45]. Thus, cytotoxic radicals such as ●OH must be neutralized without compromising the essential biological activities of other ROS, including NO• [46, 47]. 

## 3 CHARACTERISTICS OF MOLECULAR HYDROGEN

We found that H_2_ functions as a mild but effective antioxidant [12]. Hydrogen is the most abundant element in the universe, constituting nearly 75% of the universe's mass; however, hydrogen is absent on the earth in its monoatomic form and is present in water and organic or inorganic compounds. Hydrogen gas, with the molecular formula H_2_, is a colorless, odorless, tasteless and highly combustible diatomic gas. The earth's atmosphere contains less than 1 part per million of hydrogen gas [48]. 

H_2_ is rather less active and behaves as an inert gas in the absence of catalysts or at body temperature. H_2_ does not react with most compounds, including oxygen gas at room temperature. Hydrogen gas is flammable only at temperature higher than 527°C, and explodes by a rapid chain reaction with oxygen only in the explosive range of the H_2_ concentration (4 - 75%, vol/vol). 

H_2_ can be dissolved in water up to 0.8 mM (1.6 ppm, wt/vol) under atmospheric pressure, and rapidly H_2_ penetrates the glass and plastic walls of any vessels, while aluminum containers are able to retain hydrogen gas for a long time. 

## 4 SCAVENGING EFFECTS ON HYDROXYL RADICALS IN CULTURED CELLS 

### 4.1 Scavenging ●OH, but Not ●O_2_^-^, H_2_O_2_ and NO in Cultured Cells

H_2_ scavenges ●OH, but not ●O_2_^-^, H_2_O_2_ and NO in cultured cells. H_2_ was dissolved in culture medium under high pressure of hydrogen gas or by simply bubbling with hydrogen gas. The medium was combined with O_2_-saturated medium at the ratio of 8 : 2 (H_2_: O_2_). Hydrogen and oxygen concentrations and pH were monitored with each specific electrode. Cultured cells were treated with a mitochondrial respiratory complex III inhibitor, antimycin, A to induce excess ●O_2_^-^ production. Following such treatment, ●O_2_^-^ was rapidly converted to H_2_O_2_ and then ●OH. The addition of antimycin A actually increased levels of ●O_2_^-^ and H_2_O_2_ inside cells; however, H_2_ dissolved in culture medium did not change their levels. Additionally, H_2_ did not decrease the steady-state level of NO in cells. In contrast, H_2_ treatment significantly decreased levels of ●OH, as judged by the decrease in the fluorescent signal of hydroxyphenyl fluorescein (HPF) [49] and in the spin trap signals. Notably, H_2_ decreased ●OH levels even in the nuclear region [12].

After antimycin A treatment, H_2_ prevented the decline of the mitochondrial membrane potential. This suggested that H_2_ protected mitochondria from ●OH. Along with this protective effect, H_2_ also prevented a decrease in the cellular level of ATP synthesized in mitochondria. The fact that H_2_ protected mitochondria and nuclear DNA provided evidence that H_2_ penetrated most membranes and diffused into organelles. Consequently, H_2_ protected cultured cells against oxidative stress [12].

### 4.2 Other Effects Shown by Using Culture Systems

H_2_ dissolved in medium protected cultured auditory hair cells from free radicals [50] and is suggested to decrease ●OH, as judged by the decrease in HPF fluorescence in vestibular tissue [51].

●OH causes most ionizing radiation-induced cellular damage. H_2_ exhibited protective effects against radiation-induced damage in cultured cells and mice [52]. Cosmic radiation is known to induce DNA and lipid damage associated with increased oxidative stress and remains a major concern in space travel. It is expected that space mission activities will increase in coming years both in number and duration. It is therefore important to estimate and prevent the risks encountered by astronauts due to oxidative stress prior to developing clinical symptoms of disease. Schoenfeld *et al*. hypothesized that H_2_ administration to astronauts by either inhalation or drinking hydrogen water may potentially yield a novel and feasible preventative/therapeutic strategy to prevent radiation-induced adverse events [53].

On the other hand, H_2_ treatment prolonged the replicable lifespan of bone marrow multipotential stromal cells *in vitro* while preserving differentiation and paracrine potentials. Cell therapy with bone marrow multipotential stromal cells/mesenchymal stem cells represents a promising approach in the field of regenerative medicine. Low frequency of mesenchymal stem cells in adult bone marrow necessitates *ex vivo* expansion of mesenchymal stem cells after harvest; however, such manipulation causes cellular senescence with loss of differentiation, proliferative, and therapeutic potentials of mesenchymal stem cells. As oxidative stress is one of the key insults promoting cell senescence *in vivo* as well as *in vitro*, H_2_ prevented the senescent process during mesenchymal stem cell expansion. Notably, 3% hydrogen gas treatment did not decrease ●OH, protein carbonyl, and 8-hydroxydeoxyguanosine, suggesting that scavenging ●OH might not be responsible for these effects of hydrogen gas in this study [54].

## 5 ADVANTAGES OF HYDROGEN

### 5.1 Rapid Diffusion

H_2_ has a number of advantages as a potential antioxidant. First, it has favorable distribution characteristics with its own physical ability to penetrate biomembranes and diffuse into the cytosol. 

Excessive oxidative damage is a major factor because the mitochondrial respiratory chain is a significant source of damaging reactive oxygen species; however, despite the clinical importance of mitochondrial oxidative damage, antioxidants have been of limited therapeutic success. This may be because antioxidants are not selectively taken up by mitochondria [55-57]. As H_2_ effectively reaches the nucleus and mitochondria, the protection of nuclear DNA and mitochondria suggests preventive effects on lifestyle-related diseases, cancer and the aging process [12]. Moreover, H_2_ passes through the blood brain barrier, although most antioxidant compounds cannot; this is also an advantage of H_2_. 

Monitoring H_2_ concentration inside various tissues can prove gaseous diffusion [58].

### 5.2 No Direct Elimination of Functionally Important ROS

Despite their cytotoxic effects, low concentrations of ROS, such as ●O_2_^-^ and H_2_O_2_, function as signaling molecules and regulate apoptosis, cell proliferation, and differentiation [14, 15]. As mentioned, unexpectedly and notably, recent studies have suggested that excessive antioxidants increased mortality and rates of cancer [9, 11, 59-62] because they may interfere with some essential defensive mechanisms [13, 60, 63-67]. At higher concentrations, H_2_O_2_ is converted to hypochlorous acid by myeloperoxidase to defend against bacterial invasion [68]. Additionally, NO functions as a neurotransmitter and is essential for the dilation of blood vessels [69].

Since H_2_ reduces ●OH but does not affect ●O_2_^-^ and H_2_O_2_ having physiological roles [12], we propose that the adverse effects of H_2_ are very small compared to other antioxidants. 

### 5.3 No toxicity Even at Higher Concentration

Several medical gasses are expected to provide more effective therapeutic interventions and preventive medicine despite their severe toxicity. Gas inhalation as disease therapy has received recent interest [70]. In past decades, there has been extraordinary, rapid growth in our knowledge of gaseous molecules, including nitric oxide (NO), carbon monoxide (CO), and hydrogen sulfide (H_2_S), which have been known to play important roles in biological systems [71, 72].

In pre-clinical experimental models of disease, including ischemia-reperfusion injury, the inhalation of exogenous CO or H_2_S has produced a favorable outcome for most vital organs [73-76]. In particular, NO has been approved as a therapeutic agent in clinical practice [77]. The inherent toxicity of these gasses must be investigated for gas inhalation to be considered an effective therapeutic strategy because these gasses are highly toxic at considerable concentrations. Additionally, NO enhances oxidative stress via the reaction with ●O_2_^–^ by the production of highly oxidative peroxynitrite (NO + ●O_2_^–^ → ONOO^–^). It is unknown if the therapeutically effective threshold for CO or H_2_S can be attained locally in target organs without delivering a potentially toxic level of the gasses via the lungs. 

In contrast, H_2_ has more advantages from the aspect of toxicity: H_2_ has no cytotoxicity even at high concentration [78-81]. Furthermore, safety standards have been established for high concentrations of hydrogen gas for inhalation since high pressure hydrogen gas is used in deep diving gas mixes to prevent decompression sickness and arterial gas thrombi [81]. The safety of H_2_ for humans is demonstrated by its application in Hydreliox, an exotic, breathing gas mixture of 49% H_2_, 50% helium and 1% O_2_, which is used to prevent decompression sickness and nitrogen narcosis during very deep technical diving [78-81]. 

## 6 METHODS OF INGEST HYDROGEN I: INHALATION OF HYDROGEN GAS

### 6.1 Inhalation of Hydrogen Gas

Inhalation of hydrogen gas is a straightforward therapeutic method. Hydrogen gas can be inhaled by delivering hydrogen gas through a ventilator circuit, facemask or nasal cannula. Since inhaled hydrogen gas acts more rapidly, it may be suitable for defense against acute oxidative stress. In particular, inhalation of gas does not affect blood pressure [12]; blood pressure increased by infusion may cause serious obstacles during the treatment of myocardial infarction. Hydrogen gas poses no risk of explosion in air and in pure oxygen when present at concentrations < 4%, as mentioned earlier; however, safety could be a concern and the desired concentration of H_2_ must be monitored and maintained with an approved and commercially available tool.

Rats inhaled hydrogen gas in a mix of nitrous oxide (N_2_O) (for anesthesia), O_2_, and N_2_. The inhalation of H_2_ actually increased H_2_ dissolved in arterial blood depending upon the hydrogen gas concentrations, and H_2_ levels in venous blood were lower than in arterial blood; the different level between arterial and venous blood indicates the amount of H_2_ incorporated into tissues [12].

### 6.2 Direct Demonstration of Rapid Diffusion of Hydrogen as a Medical Gas

Gasses possess the ability to diffuse readily in different materials and become uniformly distributed within a defined space. “Biologic gasses” are assumed to diffuse freely across biologic membranes, acting in a variety of functional capacities [70]; hydrogen gas is an example of this.

The gaseous diffusion of H_2_ is indeed proven by monitoring its concentration inside various tissues. H_2_ can be detected with specific electrodes. H_2_ concentration has been monitored within a rat myocardium. The electrode was inserted into the non-ischemic myocardium of the left ventricle. The incremental rate of H_2_ saturation for the non-ischemic myocardium and arterial blood was similar. Then, the electrode was inserted into the ‘at risk’ area for infarction to investigate the diffusion of H_2_ into the ischemic myocardium, induced by coronary artery occlusion. Notably, H_2_ concentration was increased even in the ischemic myocardium. Although the incremental rate of H_2_ saturation was slower in the ischemic myocardium than in the non-ischemic myocardium, the peak level of H_2_ in the ischemic myocardium was approximately two thirds of the value observed for the non-ischemic myocardium [58].

### 6.3 Protective Effects on Ischemia Reperfusion Model by Rat Cerebral Infarction 

Hydrogen gas was applied to a rat model of ischemia-reperfusion as an acute model [82]. We produced focal ischemia by occlusion of the rat middle cerebral artery with subsequent reperfusion. One day after middle cerebral artery occlusion, infarct volumes decreased in a H_2_-dependent manner. One week after middle cerebral artery occlusion, the difference in infarct volumes between non-treated and H_2_-treated rats increased. H_2_-treated rats also showed improvements in body weight and temperature and movement defects vs. untreated rats. Thus, H_2_ suppressed not only the initial brain injury, but also the progression of injury. H_2_ markedly decreased several oxidative stress markers. In this experiment, H_2_ was demonstrated to have the potential to markedly decrease oxidative stress and suppress brain injury [12].

### 6.4 Protective Effects on Hepatic and Cardiac Ischemia Reperfusion Injury

Next, inhalation of hydrogen gas was also applied to a hepatic ischemia reperfusion injury model [83]. Inhalation of H_2_ clearly attenuated the degeneration induced by hepatic ischemia reperfusion and increased the protective effect in an H_2_-dependent manner. In contrast, helium gas (He) exhibited no effect, indicating that H_2_ clearly has a specific protective effect [84].

The degree of cardioprotection against ischemia-reperfusion injury was evaluated by measuring oxidative damage and infarct size after left anterior descending coronary artery occlusion and reperfusion. Inhalation of an incombustible level of hydrogen gas (2%) before reperfusion significantly reduced oxidative stress-induced myocardial injury and infarct size without affecting hemodynamic parameters, and thereby prevented deleterious left ventricle remodeling [58].

### 6.5 Protective Effects in Organ Transplantation

H_2_ inhalation significantly ameliorated intestinal and pulmonary transplant injury and prevented remote organ inflammation via its antioxidant effects [85, 86]. Ischemia/reperfusion injury during small intestinal and lung transplantation frequently causes complications, including dysmotility, inflammation and organ failure. 

H_2_ treatment resulted in significantly improved gastrointestinal transit, as well as jejunal smooth muscle contractility in response to bethanechol [86]. Graft lipid peroxidation was significantly reduced in the presence of H_2_, demonstrating antioxidant effects of H_2_ in the transplanted lungs. Exposure to 2% hydrogen gas significantly blocked the production of several pro-inflammatory mediators and reduced apoptosis with induction of the anti-apoptotic molecules B-cell lymphoma-2 and B-cell lymphoma-extra large. 

Rat cardiac cold ischemia reperfusion injury was ameliorated with inhaled H_2_ or carbon monoxide (CO), or both. Combined therapy with H_2_ and CO demonstrated enhanced therapeutic efficacy via both anti-oxidant and anti-inflammatory mechanisms, and may be a clinically feasible approach for preventing cold ischemia reperfusion injury of the myocardium [87]. Inhaled hydrogen gas effectively reduced ventilator-induced lung injury-associated inflammatory responses, at both a local and systemic level, via its antioxidant, anti-inflammatory and anti-apoptotic effects [88].

### 6.6 Protective Effects in Infectious Diseases and anti-inflammatory Effects 

Sepsis, a multiple organ dysfunction syndrome, is the leading cause of death in critically ill patients [89]. Hydrogen gas inhalation significantly improved the survival rate and organ damage of septic mice with moderate or severe cecal ligation and puncture by reducing levels of early and late pro-inflammatory cytokines in serum and tissues [90].

The effects of 2% H_2_ treatment was investigated on the survival rate and organ damage in zymosan-induced generalized inflammation model. The beneficial effects of H_2_ treatment zymosan-induced organ damage were associated with decreased levels of oxidative product, increased activities of antioxidant enzyme, and reduced levels of early and late pro-inflammatory cytokines in serum and tissues. H_2_ treatment protected against multiple organ damage in a zymosan-induced generalized inflammation model, suggesting the potential use of H_2_ as a therapeutic agent in the therapy of conditions associated with inflammation-related multiple organ dysfunction syndrome [91].

### 6.7 Others

Other reports had the following titles: Hydrogen therapy reduces apoptosis in neonatal hypoxia-ischemia rat model [92]; hydrogen gas reduced acute hyperglycemia-enhanced hemorrhagic transformation in a focal ischemia rat model [93]; hydrogen is neuroprotective and preserves cerebrovascular reactivity in asphyxiated newborn pigs [94]; beneficial effects of hydrogen gas in a rat model of traumatic brain injury via reducing oxidative stress[95]; beneficial effects of hydrogen gas against spinal cord ischemia-reperfusion injury in rabbits [96]; and hydrogen protects vestibular hair cells from free radicals [97].

## 7 METHODS OF INGEST HYDROGEN II: ORAL INGESTION OF HYDROGEN WATER 

### 7.1 Oral Ingestion by Drinking Hydrogen Water

Since inhaled hydrogen gas acts more rapidly, it may be suitable for defense against acute oxidative stress. In particular, inhalation of gas does not affect blood pressure; blood pressure increased by infusion may be serious in myocardial infarction; however, inhalation of hydrogen gas may be unsuitable or not practical as continuous H_2_ consumption in daily life for preventive use. In contrast, solubilized H_2_ (H_2_-dissolved water; namely, hydrogen water) may be beneficial since it is a portable, easily administered and a safe means of delivering H_2_ [98]. H_2_ can be dissolved in water up to 0.8 mM under atmospheric pressure at room temperature as mentioned earlier. Unexpectedly, drinking hydrogen water had effects comparable to hydrogen gas inhalation [99]. 

Hydrogen water can be made by several methods, including dissolving hydrogen gas in water under high pressure, dissolving electrolyzed H_2_ in water, and by the reaction of magnesium metal with water. The method of dissolving hydrogen gas under high pressure has an advantage because it is applicable not only using water but also any other solvents. 

When water saturated with H_2_ was placed into the stomach of a rat, H_2_ was detected at several µM level in blood [98, 99]. Moreover, hepatic H_2_ was monitored with a needle-type hydrogen electrode, and H_2_ accumulated after oral administration of hydrogen water, partly explaining why consumption of even a small amount of H_2_ over a short dwell time could efficiently improve various disease models. An additional *in vitro* experiment confirmed that polymers of carbohydrates, including glycogen and starch, have an affinity for H_2_ [99]. 

### 7.2 Prevention of Cognitive Decline

Chronic physical restraint stress on mice enhanced levels of oxidative stress in the brain, and impaired learning and memory [100, 101]. Consumption of hydrogen water *ad libitum* suppressed the increase in oxidative stress, and prevented cognitive impairment. Neural proliferation in the dentate gyrus of the hippocampus was suppressed by restraint stress [101]. The consumption of hydrogen water ameliorated the reduced proliferation; however, a mechanistic link between H_2_-dependent changes in neurogenesis and cognitive impairments remains unclear. Thus, continuous consumption of hydrogen water reduced oxidative stress in the brain and prevented the stress-induced decline in learning and memory [98]. 

### 7.3 Preventive and Therapeutic Affects on Parkinson Disease Model

In Parkinson’s disease, mitochondrial dysfunction and the associated oxidative stress are major causes of dopaminergic cell loss in the substantia nigra [102]. H_2_ in drinking water was given before or after stereotactic surgery for 6-hydroxydopamine-induced nigrostrital degeneration in a rat model of Parkinson’s disease. Hydrogen water prevented both the development and progression of nigrostriatal degeneration. Hydrogen water likely retards the development and progression of Parkinson’s disease [103]. 

Drinking hydrogen water suppressed dopaminergic neuronal loss in another Parkinson’s disease model induced by MPTP (1-methyl-4-phenyl-1,2,3,6-tetrahydropyridine) [104].

### 7.4 Prevention of Atherosclerosis Model

Oxidative stress is involved in atherosclerosis [105, 106]; however most clinical trials of dietary antioxidants failed to show marked success in preventing atherosclerotic diseases [8, 107, 108]. Drinking hydrogen water *ad libitum* decreased the aorta oxidative stress level and prevented arteriosclerosis in an apolipoprotein E knockout mouse [109]. Thus, consumption of hydrogen water has potential to prevent arteriosclerosis more effectively than other antioxidants [110].

### 7.5 Improvement of Metabolic Syndrome

Increased oxidative stress in obesity affects metabolic syndrome [111]. Long-term drinking of hydrogen water significantly controlled fat and body weights, despite no change in the consumption of food and water. Moreover, drinking hydrogen water decreased levels of plasma glucose, insulin and triglyceride, the effect of which on hyperglycemia was similar to diet restriction [112]. A mechanistic study revealed that the gene expression of the hepatic hormone, fibroblast growth factor 21 (FGF21) was enhanced, which should function to enhance fatty acid and glucose expenditure. Indeed, drinking hydrogen water stimulated energy metabolism, as measured by O_2_ consumption and CO_2_ expiration. These results suggest the potential benefit of H_2_ in improving obesity, diabetes and metabolic syndrome [112].

### 7.6 Prevention of Adverse Effects by an Anti-tumor Drug

Cisplatin is a widely used anti-cancer drug in the treatment of a wide range of tumors; however, its application is limited by causing nephrotoxicity, which may be mediated by oxidative stress [113]. Inhalation of hydrogen gas (1% H_2_ in air) or drinking hydrogen water improved mortality and body-weight loss caused by cisplatin, and alleviated nephrotoxicity. Consumption of hydrogen water improved metamorphosis accompanying decreased apoptosis in the kidney. Despite its protective effects against cisplatin-induced toxicity, H_2_ did not impair the anti-tumor activity of cisplatin against cancer cell lines *in vitro* and in tumor-bearing mice *in vivo*. Thus, H_2_, whether hydrogen gas or hydrogen water, could improve the quality of life of patients during chemotherapy [99]. This finding was confirmed by another group [114].

### 7.7 Anti-allergic Reactions

It was demonstrated using a mouse model that drinking hydrogen water could attenuate an immediate-type allergic reaction by suppressing the phosphorylation of FcεRI-associated Lyn and its downstream signaling molecules, which subsequently inhibited NADPH oxidase activity and reduced the generation of hydrogen peroxide [115]. These findings imply that the beneficial effects of H_2_ are not only imparted by its radical scavenging activity, but also by modulating a specific signaling pathway.

### 7.8 Effects on Transplantation

ROS contributes to the development of interstitial fibrosis and tubular atrophy seen in chronic allograft nephropathy. Nakao’s group tested the effect of treatment with hydrogen water in a model of kidney transplantation, in which allografts from Lewis rats were orthotopically transplanted into Brown Norway recipients that had undergone bilateral nephrectomy. Drinking hydrogen water improved allograft function, slowed the progression of chronic allograft nephropathy, reduced oxidant injury and inflammatory mediator production, and improved overall survival. Inflammatory signaling pathways, such as mitogen-activated protein kinases, were less activated in renal allografts from hydrogen water-treated rats as compared with normal water-treated rats. Thus, oral hydrogen water is an effective antioxidant and anti-inflammatory agent that reduced chronic allograft nephropathy, improving the survival of rat renal allografts [116].

### 7.9 Others

It has been shown that drinking hydrogen water prevents superoxide formation in brain slices of vitamin C-depleted SMP30/GNL knockout mice [117], that H_2_ in drinking water attenuates noise-induced hearing loss in guinea pigs [118], that drinking hydrogen water ameliorated cognitive impairment in senescence-accelerated mice [119], and that H_2_ exhibited potential cardioprotective effects in irradiated mice [120].

## 8 METHODS OF INGEST HYDROGEN III: INJECTION OF HYDROGEN SALINE

### 8.1 Advantage of injection

Even though oral administration is safe and convenient, H_2_ in water tends to escape over time and some H_2_ is lost in the stomach or intestine, making it difficult to control the concentration of H_2_ administered. Administration of H_2_ via an injectable hydrogen saline (H_2_-dissolved saline) vehicle may allow the delivery of more accurate concentrations of H_2_ [121].

### 8.2 Effects of Hydrogen Saline on Various Disease Models

Sun’s group administered H_2_-saturated saline by peritoneal injection to various model animals with great success. Thus, hydrogen saline has potential in actual clinical treatment. For example, injection of hydrogen saline showed neuroprotective effects in a neonatal hypoxia-ischemia rat model [121]. Moreover, H_2_ saline was applied to an Alzheimer’s disease model mouse, which was generated by intracerebroventricular injection of the Aβ1-42 peptide. H_2_ treatment decreased the level of oxidative stress and inflammation markers and prevented memory dysfunction and motor dysfunction, respectively [122].

They and other groups have demonstrated effects on many disease models, as published in the following reports [123-130]. 

## 9 METHODS OF INGEST HYDROGEN IV: DIRECT ABSORPTION OF HYDROGEN 

### 9.1 Improvement of Glaucoma Model

Alternatively, H_2_-loaded eye drops were prepared by dissolving H_2_ in saline and directly administering to the ocular surface [131, 132].

In acute glaucoma of the eyes, transient elevation of intraocular pressure causes significant reductions in the thickness of the retina by ischemia-reperfusion injury mediated through the generation of reactive oxygen species [133]. The direct application of eye drops containing H_2_ ameliorated ischemia-reperfusion injury of the retina in a rat model. When H_2_ eye drops were continuously administered, the H_2 _concentration increased in the vitreous body and the •OH level decreased during retinal ischemia-reperfusion. H_2 _eye drops reduced the number of apoptotic and oxidative stress marker-positive cells 1 day after ischemia-reperfusion injury, and reduced retinal thinning with accompanying activation of Müller glia, astrocytes and microglia at 7 days after ischemia-reperfusion injury, improving the recovery of inner retinal layer thickness to >70%.

Moreover, we devised eye drops with dissolved H_2_ to directly administer H_2_ to the retina, and monitored the time course of changes in H_2 _levels using a needle-shaped hydrogen sensor electrode inserted through the sclera to the vitreous body in rats. H_2_ was able to reach the vitreous body by administering H_2_ saturated in normal saline. When H_2_ eye drops were administered continuously, approximately 70% H_2_ was detected on the ocular surface. Two minutes after the start of administration, H_2 _concentration in the vitreous body started to increase and reached a maximum level after 15 min. At that time, H_2_ concentration was approximately 20% of H_2_ in the eye-drops. The maximum concentration of H_2 _in the vitreous body reached approximately one third of the value observed on the ocular surface [131].

### 9.2 Hydrogen Bath

H_2_ easily penetrates the skin and distributes throughout the body via blood flow. Thus, taking a warm water bath with dissolved H_2_ is a method of incorporating H_2_ into the body in daily life, especially in Japan. It takes only 10 minutes to distribute throughout the whole body, as judged by measuring hydrogen gas in expiration (unpublished results). 

## 10 METHODS OF INGEST HYDROGEN V: INCREASE IN INTESTINAL HYDROGEN

### 10.1 Production of Hydrogen in Intestinal Bacteria

Other medical gasses, CO, NO and H_2_S, are generated by endogenous enzymatic systems. Pharmaceutical development has taken advantage of these systems to design exogenous molecules to simulate those generated endogenously; however, mammals lack their own enzyme to produce H_2_ [70]. 

Instead of endogenous enzymatic systems, the spontaneous production of hydrogen gas in the human body occurs via the fermentation of undigested carbohydrates by resident enterobacterial flora [134]. H_2_ is transferred to the portal circulation and excreted through the breath in significant amounts [135]. For this reason, measurement of H_2_ levels in expired air is used to detect carbohydrate malabsorption [76]; however, there have been few studies on the physiological function of gastrointestinal tract-derived hydrogen gas as an antioxidant. 

### 10.2 Are α-glucosidase Inhibitors an Indirect Antioxidant?

α-Glucosidase inhibitors are pharmacological agents that specifically reduce postprandial hyperglycemia through retardation of disaccharide digestion, thereby reducing glucose absorption. A large scale epidemiologic trial has demonstrated that the treatment of patients with impaired glucose tolerance with an α-glucosidase inhibitor was associated with a 25% reduction in the risk of progression to diabetes, a 34% reduction in the risk of developing de novo hypertension, and a 49% risk reduction of cardiovascular events [136]. Furthermore, meta-analysis of seven long-term studies suggested that acarbose reduced the risk of myocardial infarction for patients with type 2 diabetes [137]. Such risk reduction for coronary heart disease events in patients with type 2 diabetes was not observed by improved glycemic control achieved by intensified treatment with insulin and glibenclamid [138]. Actually, acarbose, which is an α-glucosidase inhibitor, markedly increased H_2_ production in volunteers. Thus, we propose that H_2_ produced by intestinal bacteria acts as a unique antioxidant and prevents cardiovascular events [139].

### 10.3 Anti-inflammation Effects by Intestinal Bacteria via Hydrogen

*	Escherichia coli* can produce a considerable amount of H_2_ by catalyzing with hydrogenase. Kawai *et al*. examined whether H_2_ released from intestinally colonized bacteria could affect concanavalin A-induced mouse hepatitis. Reconstitution of intestinal flora with H_2_-producing *E. coli*, but not hydrogenase-deficient mutant *E. coli*, down-regulated concanavalin A-induced liver inflammation. These results indicate that H_2_ released from intestinal bacteria can suppress inflammation [140]. H_2_ also mediates the suppression of colon inflammation induced by dextran sodium sulfate [141].

### 10.4 Others

Dietary turmeric induced H_2_ production from the intestinal bacteria [142], and lactulose was shown to be an indirect antioxidant ameliorating inflammatory bowel disease [143].

## 11 CLINICAL TESTS

Several groups have started clinical examinations. Clinical tests have revealed that drinking hydrogen water reduced oxidative stress markers in patients with type 2 diabetes [144] or subjects with potential metabolic syndrome [145] and influenced glucose [144] and cholesterol metabolism [145]. 

Hemodialysis using dialysis solution with H_2_ significantly decreased the levels of plasma monocyte chemoattractant protein 1 and myeloperoxidase [146]. 

## 12 REGULATION OF GENE EXPRESSIONS AND PROTEIN PHOSPHORYLATIONS

It has been reported that H_2_ acts as an anti-inflammatory and anti-allergic regulator by inducing inflammatory cytokines and inhibiting phosphorylating signal factors, respectively; however, the transcriptional factors and kinases involved in the effects afforded by H_c_ have not been identified. 

H_2_ decreased the expressions of pro-inflammatory factors, including TNF-α, IL-6, IL-1β, CCL2 and IL-10, TNF-γ, IL-12, ICAM-1 [85], HMGB-1 [147], NF-κB [148], PGE2, and PGE2 [54].

Moreover, H_2_ up- or down-regulated the factors involved in apoptosis toward the inhibition of apoptosis: H_2_ suppressed the expressions of pro-apoptotic factors, including casapase 3 [92, 149], and caspase 12 [92], caspase 8 [86] and BAX [86]. Conversely H_2_ stimulated the expressions of the anti-apoptoptic factors of Bcl-2 and Bcl-xL [86].

H_2_ is involved in the regulation of various factors; up-regulation of PCNA, bFGF, HGF, IFN-γ, and down-regulation of i-NOS [87] and VEGF [54]. 

As a signal transduction contributor, H_2_ inhibited the phospahorylations of some signal proteins, including MEK, p38, ERK, JNK [116] and Lyn, Syk, PLCγ1, γ2, Akt, ERK1/2, JNK, p38, cPLA2, ASK1, IκBα [115].

Heme oxygenase-1 (HO-1), a microsomal enzyme degrading heme to carbon monoxide, free iron, and biliverdin, participates in the cell defense against oxidative stress and has been speculated to be a new therapeutic target [150]. Notably, H_2_ modulates HO-1 expression, which is commonly up-regulated by these medical gasses [48, 151]. Additionally, H_2_ up-reguated the expression of FGF21, which is a regulator of energy metabolism [112].

As essential questions, it remains unknown how H_2_ regulates gene expressions and phosphorylations, and whether the above regulations of transcription and phosphorylation are the cause or consequence of the effects of H_2_. The primary molecular target of H_2_ remains unknown. 

## 13 CLOSING REMARKS: ISSUES TO BE DISSOLVED IN THE FUTURE

In our first report published in 2007, we indicated that H_2_ reacted with strong reactive oxygen/nitrogen species, including ●OH and ONOO^–^ in cell-free reactions. Cells cultured in H_2_-rich medium were protected against oxidative stress by the ●OH-scavenging activity of H_2_, depending upon the decrease of ●OH [12]; however, recent evidence shows that the scavenging property is not the only explanation for the potent beneficial effects of H_2_. When model animals and human subjects consumed H_2_ by drinking water with dissolved H_2_, even a very small amount of H_2_ was extensively effective. It may be difficult to explain that direct reduction of ●OH by a very small amount of H_2_ reveals all the functions of H_2_, because the saturated level of H_2_ is only 0.8 mM and the dwelling time of ●OH is very short in the body.

We have recently shown that H_2_ can be accumulated with hepatic glycogen; this finding indicates the possible accumulation of H_2_ in a specific region; however, it is unlikely that the amount of H_2_ is sufficient to exhibit all of its functions [112]. Additionally, drinking 0.04 or 0.08 mM H_2_ was shown to be effective [104, 112]. The amount of administered H_2_ seems to be, in many cases, independent of the magnitude of effects. Intestinal bacteria produce more than 1 liter of hydrogen gas per day, whereas the amount of H_2_ originating from drinking hydrogen water is less than 50 ml. Nevertheless, additional H_2_ in drinking hydrogen water is unambiguously effective.

Many additional issues of hydrogen therapy including the molecular mechanisms underlying the marked effects of a very small amount of H_2_ remain elusive. The primary molecular target of H_2_ remains unknown. Although H_2_ regulates various gene expressions and protein-phosphorylations, it remains unclear whether such regulations are the cause or consequence of the effects against oxidative stress. One of the open questions is how H_2_ involves the cross-talk among anti-oxidation, anti-inflammation and anti-allergy. Thus, it should not be fair to classify the roles of H_2_ by outward effects at this stage.

Finally, the author summarizes the reports showing the effects of H_2_ by the classification of target organs (Table **1**) [152].

## Figures and Tables

**Fig. (1):**
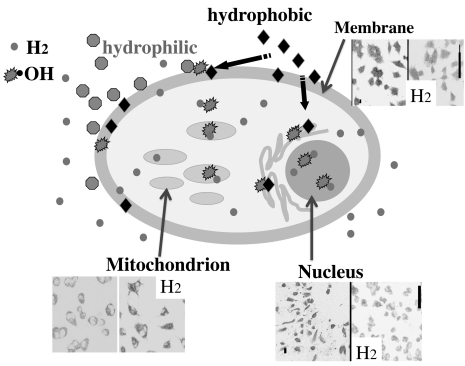
**Illustration of gaseous diffusion of H_2_ in a cell**. Most hydrophilic compounds (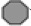) retain at membranes and cannot reach the cytosole, whereas most hydrophic ones (♦) cannot penetrate biomembranes in the absence of specific carriers. In contrast, H_2_ (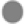) can rapidly distribute into cytosol and organelles. PC12 cells were placed in culture media containing H2 (0.6 mM) and O_2_ (0.24 mM), and then oxidative stress was induced by adding antimycin A (10 µg/mL), an inhibitor of the electron transport chain of mitochondria, and maintained for 1 day. Two markers of oxidative stress were detected by immunostaining with anti-8-hydroxy-Guanine (**Nucleus**) and anti-4-hydoroxy-2-nonenal (**Membrane**). Thirty minutes after adding antimycin A with or without H_2_, 100 nM tetramethylrhodamine methyl ester (TMRM), a fluorescent detector of the membrane potential of **mitochondrion**, were added, incubated for 10 min, and cells were imaged with a laser scanning confocal microscope. These results indicate that H_2_ reach the nucleus and mitochondria and protects them.

**Fig. (2). Measurement of the accumulation of H2 in rat liver.**
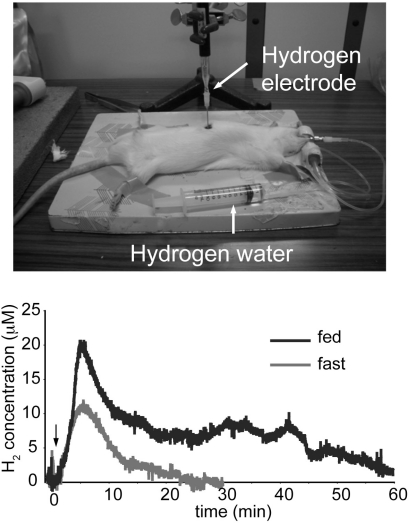
The concentration of H_2_ in the liver was monitored using a needle-type hydrogen sensor inserted into fed- or overnight fasted-rat liver. Rat received hydrogen water (0.8 mM H_2_ in water) orally by stomach gavage at 15 ml/kg. Arrow indicates the time point when rat was administered hydrogen water.

**Table 1.** Diseases and Physiological States for Which Hydrogen Effects are Reported as Classified by Target Organs [152].

Disease/Physiology	Species	Source of H_2_	Reference
**Brain**			
Cerebral infarction	rodent	gas	[12]
Superoxide in brain	rodent	water	[117]
Neonatal brain hypoxia	rodent	gas	[92]
	rodent	saline	[131]
	pig	gas	[94]
Restraint-induced dementia	rodent	water	[98]
Alzheimer’s disease	rodent	saline	[122]
Senile dementia	rodent	water	[119]
Parkinson’s disease	rodent	water	[103, 104]
Hemorrhagic cerebral infarction	rodent	gas	[93]
Traumatic brain injury	rodent	gas	[95]
**Spinalcord**			
Spinal cord injury	rodent	saline	[130]
**Eye**			
Glaucoma	rodent	eye drops	[131]
Corneal alkali burn	rodent	eye drops	[132]
**Ear**			
Hearing disturbance	rodent	medium	[50]
	rodent	gas	[97]
	rodent	water	[118]
**Lung**			
Oxygen-induced lung injury	rodent	saline	[128, 129]
Lung transplantation	rodent	gas	[86]
**Heart**			
Myocardial infarction	rodent	gas	[58]
	rodent	saline	[149]
Heart transplantation	rodent	gas	[87]
Irradiation-induced heart injury	rodent	water	[120]
**Liver**			
Hepatic ischemia	rodent	gas	[84]
Hepatitis	rodent	bacteria	[140]
Obstructive jaundice	rodent	saline	[124]
**Kidney**			
Cisplatin nephropathy	rodent	gas, water	[99]
	rodent	water	[114]
Hemodialysis	human	dialysis	[146]
Kidney transplantation	rodent	water	[116]
**Pancreas**			
Acute pancreatitis	rodent	saline	[148]
**Intestine**			
Intestinal graft	rodent	gas	[85]
	rodent	saline	[125, 130]
Ulcerative colitis	rodent	gas	[141]
**Bloodvessel**			
Atherosclerosis	rodent	water	[110]
**Metabolism**			
Diabetes mellitus type 2	human	water	[144]
Metabolic syndrome	human	water	[145]
Obesity/Diabetes	rodent	water	[112]
**Inflammation and allergy**			
Allergy type I	rodent	water	[115]
Sepsis	rodent	gas	[90]
Zymosan-induced inflammation	rodent	gas	[91]
**Others**			
Multipotent stromal cells	cells	medium	[54]
Radiation injury	cells, rodent	medium	[52]
